# β-cryptoxanthin suppresses oxidative stress via activation of the Nrf2/HO-1 signaling pathway in diabetic kidney disease

**DOI:** 10.3389/fphar.2024.1480629

**Published:** 2024-11-15

**Authors:** Jingjing Ke, Hualong Zang, Yang Liu, Qiuping Teng, Jiao Hua, Dan Peng, Ping Wang

**Affiliations:** ^1^ Department of Nephrology, Jingmen Central Hospital, Hubei Minzu University, Jingmen, Hubei, China; ^2^ Department of Nephrology, Jingmen Central Hospital Affiliated to Jingchu University of Technology, Jingmen, Hubei, China; ^3^ Department of Neonatology, Jingmen Central Hospital Affiliated to Jingchu University of Technology, Jingmen, Hubei, China

**Keywords:** β-cryptoxanthin, diabetic kidney disease, podocyte, oxidative stress, Nrf2

## Abstract

**Objectives:**

This study aims to explore the role and investigate mechanisms of β-Cryptoxanthin (BCX) in high glucose (HG)-induced podocyte injury and renal dysfunction.

**Methods:**

In this study, db/db mice were orally treated with BCX. Blood glucose, body weight, urinary albumin creatinine ratio (ACR) were recorded to evaluate the mice renal function. The H&E, PAS staining, and transmission electron microscopy (TEM) were utilized to examine the effect of BCX on the morphological changes of glomeruli in db/db mice. In addition, reactive oxygen species (ROS) content, mitochondrial membrane potential (MMP) level, ATP level, and SA-β-gal staining were used to assess the podocyte oxidative damage, mitochondrial dysfunction and senescence. Furthermore, the effects of BCX on Nrf2/HO-1 signaling pathway were evaluated *in vivo* and *in vitro* through Western blotting, immunohistochemistry and immunofluorescence analysis.

**Results:**

*In vivo*, BCX reversed glomerular mesangial matrix expansion and reduced proteinuria in db/db mice, as well as decreased glomerular oxidative stress and kidney aging. Similarly, *in vitro* study showed that BCX effectively alleviated the oxidative stress, mitochondrial dysfunction, and senescence induced by HG in podocytes. Furthermore, we identified that the antioxidative effects of BCX are associated with the activation of Nrf2/HO-1 signaling pathway, and that Nrf2 knockdown partially abrogated the protective effects of BCX *in vitro*.

**Conclusion:**

Our study demonstrated for the first time that BCX alleviates podocyte injury in DKD by promoting Nrf2/HO-1 signaling pathways. BCX may be a potential candidate compound for preventing Diabetic kidney disease (DKD).

## 1 Introduction

Diabetic kidney disease (DKD) is the leading cause of end-stage renal disease (ESRD) (Rayego-Mateos et al., 2023). Current interventions, including hypoglycemic agents, sodium-glucose cotransporter protein 2 (SGLT2) inhibitors, and renin-angiotensin-aldosterone system (RAAS) blockers are not able to completely prevent DKD progress to ESRD (Dai et al., 2023). Therefore, it is imperative to explore the underlying pathogenesis of DKD and to identify more effective intervention targets. Podocytes constitute the last layer of the glomerular filtration barrier through their foot processes (Li et al., 2023). Numerous studies have confirmed that podocyte injury is closely related to the development of DKD (Lizotte et al., 2023; Salemkour et al., 2023). Thus, elucidating the mechanisms underlying podocyte injury is crucial for developing effective treatments for DKD.

In recent years, oxidative stress has been closely linked to podocyte injury and the development of DKD (Chae et al., 2023; Mohandes et al., 2023). Under physiological conditions, superoxide dismutase (SOD), the glutathione system, and catalase play vital roles in the intracellular defense against reactive oxygen species (ROS), helping to maintain redox balance within the cell (Jomova et al., 2023). However, oxidative stress arises when the production of cellular ROS exceeds the clearance capacity of the antioxidant defense systems (Jomova et al., 2023). Moreover, excessive ROS production can lead to mitochondrial dysfunction by damage in proteins, DNA, and lipids (Su et al., 2023). Oxidative stress is regarded as a major factor in DKD, and its excessive production under diabetic conditions also contributes to podocyte injury (Su et al., 2023). Notably, numerous studies have established a close relationship between oxidative stress and cellular senescence (Li et al., 2024). Senescent cells are known to be present at increased levels in many renal diseases, and it appears that senescence plays a role in maladaptive kidney repair contributing to glomerular sclerosis and proteinuria (Lucas et al., 2023). This suggests that targeting oxidative stress and cellular senescence may be a critical strategy for inhibiting the development of DKD.

Previous studies have reported that nuclear factor erythroid 2 (NFE2)-related factor 2 (Nrf2) is a transcription factor that serves as a major regulator of cellular redox homeostasis, and has been shown to prevent kidney injury and aging (Liu et al., 2023). Nrf2 deficiency is associated with increased ROS production and apoptosis in DKD (Liu et al., 2022a). Thus, pharmacologic activation of Nrf2 has emerges as a promising therapeutic strategy for addressing podocyte injury in diabetic conditions.

Mitochondria-targeted antioxidants such as mitoquinone (MitoQ) and SS-31 (d-Arg-dimethylTyr-Lys-Phe-NH₂) have demonstrated efficacy in treating kidney diseases (Kirkman et al., 2023; Shan et al., 2023). However, the high cost of synthetic antioxidants limits their widespread use. In contrast, β-cryptoxanthin (BCX) is recognized as an antioxidant and is abundantly found in fruits and vegetables like oranges, red peppers, and pumpkins (Clugston, 2023). BCX has several important biological functions that benefit human health (Granot-Hershkovitz et al., 2023). For example, BCX can delay the progression of non-alcoholic fatty liver disease (NAFLD) by alleviating insulin resistance and oxidative stress (Clugston, 2023). Additionally, a recent study found that BCX promoted Nrf2 nuclear expression, maintained mitochondrial function to inhibit H_2_O_2_-induced oxidative stress and cellular senescence in human renal tubular epithelial (HK-2) cells, suggesting that BCX exerts a protective role by regulating the Nrf2-mediated signaling pathway (Zhang et al., 2023). Moreover, the Mikkabi Cohort Study revealed a negative correlation between BCX and the incidence of dyslipidemia and type 2 diabetes mellitus (Sugiura et al., 2015a). Therefore, we hypothesize that supplementing with BCX may be an effective strategy to protect against podocyte injury in DKD.

In this study, we investigated the protective effect of BCX on oxidative stress, mitochondrial damage and senescence in podocytes both *in vivo* and vitro, and evaluated the role of the Nrf2 pathway in this process.

## 2 Materials and methods

### 2.1 Animal studies

Eight-week-old db/db mice and matched db/m mice were purchased from CAVENS Laboratory Animals (Jiangshu, China). In animal experiments, considering that female mice are resistant to type 2 diabetes, only male mice were used (Fu et al., 2020). After 2 weeks of adaptive feeding, the mice were randomly separated into four groups: (1) db/m group (n = 6); (2) db/m + BCX (n = 6); (3) db/db group (n = 6); (4) db/db + BCX (n = 6). Mice were given BCX (10 mg/kg/d) (HY-108059, MCE, China) in a normal diet for 6 weeks (Liu et al., 2022b). Blood glucose, body weight, 24-h proteinuria and the urinary albumin creatinine ratio (ACR) were tested every 2 weeks. At the age of 16 weeks, the kidneys and blood samples were harvested for histological and biochemical analyses. All animal experimental procedures were approved by the Ethics Committee for the Experimental Use of Animals of Hubei Minzu University (No. 2021028).

### 2.2 Cell culture and treatments

Conditionally immortalized podocytes were kindly provided by Dr. Moin A. Saleem (Bristol University, Bristol, United Kingdom). Briefly, podocytes were cultured at 33°C in RPMI 1640 medium (HyClone, USA) supplemented with 10% fetal bovine serum (Gibco, USA) and 1×insulin-transferrin-selenium (ITS, Gibco) for proliferation. To differentiate, the cells were cultured at 37°C in an ITS-free medium for 7–10 days. The differentiated cells were stimulated for 24 h with high glucose (HG, 30 mM). Prior to HG exposure, the cells were pretreated with 10 μM BCX for 2 h (Zhang et al., 2023). For knockdown of Nrf2, the Nrf2 siRNA (Sangon Biotech, China) was conducted using HiPerFect (Qiagen, Germany) according to the manufacturer’s instructions.

### 2.3 SA-β-galactosidase staining

The β-galactosidase (β-gal) assay was performed using the SA-β-gal staining kit (Beyotime, C0602) according to the manufacturer’s instructions. Briefly, podocytes were fixed for 10 min, and were stained with the SA-β-gal–staining solution at pH 6.0 for 12 h. A fluorescence microscope (Leica, Germany) was used for capturing the images.

### 2.4 Transmission electron microscopy

For mice kidney tissues, transmission electron microscopy (TEM) was used for analysis. After harvesting the mice kidney tissues, they were fixed using glutaraldehyde and prepared as electron microscopic specimens. The ultrastructure of glomerular podocytes was assessed using transmission electron microscopy with randomly selected fields of view according to standard procedures.

### 2.5 Detection of mitochondrial membrane potential (MMP, △Ψm), intracellular reactive oxygen species (ROS) and ATP

The MMP of podocytes was measured by using JC-1 fluorescence probe Staining Kit (C2005, Beyotime, China); the ATP production in podocytes and kidney tissues were assayed by ATP Assay Kit (S0026, Beyotime) and relative light unit (RLU) was recorded by microplate reader. ROS production in podocytes and renal tissues was analyzed using 2′-7′-dichlorofluorescein diacetate (DCFH-DA, S0033M, Beyotime) and dihydroethidium (DHE), respectively. All procedures performed were in accordance with the manufacturer’s protocol. Fluorescence microscope (Leica, Germany) was used for capturing the images.

### 2.6 Malonaldehyde (MDA) and glutathione (GSH) detection

The reagent in the MDA detection kit (Beyotime, S0131S) was added to the cell lysis supernatant and tissue homogenates and the mixture was incubated in a water-bath at 100°C for 15 min. After cooling to room temperature, the mixture was centrifuged for 10 min 200 μL of the supernatant was transferred into a 96-well plate and the absorbance value at 532 nm was recorded using a microplate reader (EnSight; PerkinElmer, USA).

The total GSH content in podocytes and mouse kidney tissues were measured using a GSH kit (Beyotime, S0052). Briefly, the cell lysis supernatants were collected after two cycles of freeze and thaw. Then the GSH reagent was added into the supernatants or tissue homogenates. Finally, the levels of total glutathione were measured by detecting the absorbance at 412 nm.

### 2.7 Western blot analysis

The total proteins were separated by 8%–12% SDS-PAGE gels and transferred onto PVDF membranes (Millipore, USA), which were blocked with 5% bovine serum albumin (BSA) solution for 2 h at room temperature. Then, the membranes were incubated with the specific primary antibodies at 4°C overnight (Table 1). After washing with TBST (Tris buffered saline with tween 20) three times, the membranes were incubated with HRP-conjugated secondary anti-rabbit or anti-mouse antibody for 1 h. Finally, the protein bands were visualized using the ImageLab software (BioRad, USA). β-actin density was used as internal control to normalize the protein expression.

**TABLE 1:** Antibody used for Western blotting.

Source	Antibody	Dilutions	Company	Item No.
Rabbit	Nrf2	1:1,000	Proteintech	#16396-1-AP
Rabbit	HO-1	1:1,000	Proteintech	#10701-1-AP
Rabbit	Caspase3	1:1,000	CST	#9662
Mouse	Cleaved-caspase3	1:1,000	Immunoway	#YM3431
Mouse	β-actin	1:1,000	Proteintech	#66009-1-Ig
Rabbit	P16	1:1,000	Proteintech	#10883-1-AP
Rabbit	P21	1:1,000	Proteintech	#10355-1-AP
Mouse	P53	1:1,000	Proteintech	#60283-2-Ig

### 2.8 Immunofluorescence staining

Five-micrometer-thick mice kidney sections (5 μm) were deparaffinized, blocked with 5% BSA buffer, and incubated overnight with corresponding primary antibodies (WT-1, 1:100, Novus, #NBP2-44607; Nrf2, 1:50, Proteintech, #16396-1-AP; Synaptopodin, 1:100, Progen, #65194) at 4°C. After washing with TBST, the sections were then incubated in horseradish peroxidase (HRP)-conjugated or Cy3-labeled secondary antibodies for 2 h and counterstained with DAPI (1 μg/mL, Invitrogen, USA) for 10 min to visualize the nuclei.

Podocytes seeded on slides were fixed with 4% paraformaldehyde for 25 min, permeabilized with 0.1% triton X-100 (Sigma, USA) for 15 min, and blocked with 1% BSA buffer for 40 min, then the podocytes were incubated with primary antibodies against Nrf2 (Proteintech, 1:100, #16396-1-AP), or (γ-H2AX Ser139, 1:100, #ab81299) at 4 °C overnight. After washing with TBST buffer, the slides were incubated with Alex Fluro 594-conjugated secondary antibody (AntGene, 1:150, China) for 35 min. The confocal microscope (Leica, Germany) was used to visualize images.

### 2.9 Flow cytometry

Podocytes apoptosis was determined using an Annexin V-PE/7-ADD kit (BD, USA) according to the manufacturer’s instructions. Briefly, cells were seeded in six-well plates and exposed to HG and pretreated with BCX for 2 h. After digestion and centrifugation, the cells were resuspended with 100 μL binding buffer, which then supplemented with 2.5 μL Annexin V-PE and 2.5 μL 7-AAD and incubated for 20 min in the dark. The apoptotic cells were measured by CytoFLEX (Beckman, USA) and analyzed by FlowJo Software.

### 2.10 Statistical analysis

The data were expressed as the mean ± SD and were analyzed using GraphPad Prism 9.0 Software (GraphPad, USA). One-way analysis of variance (ANOVA) with Tukey’s *post hoc* test was used to compare the difference among three or more groups. *p* < 0.05 was considered to be statistically significant.

## 3 Results

### 3.1 Effects of BCX on functional and morphological characteristics in kidneys of db/db mice

**FIGURE 1:**
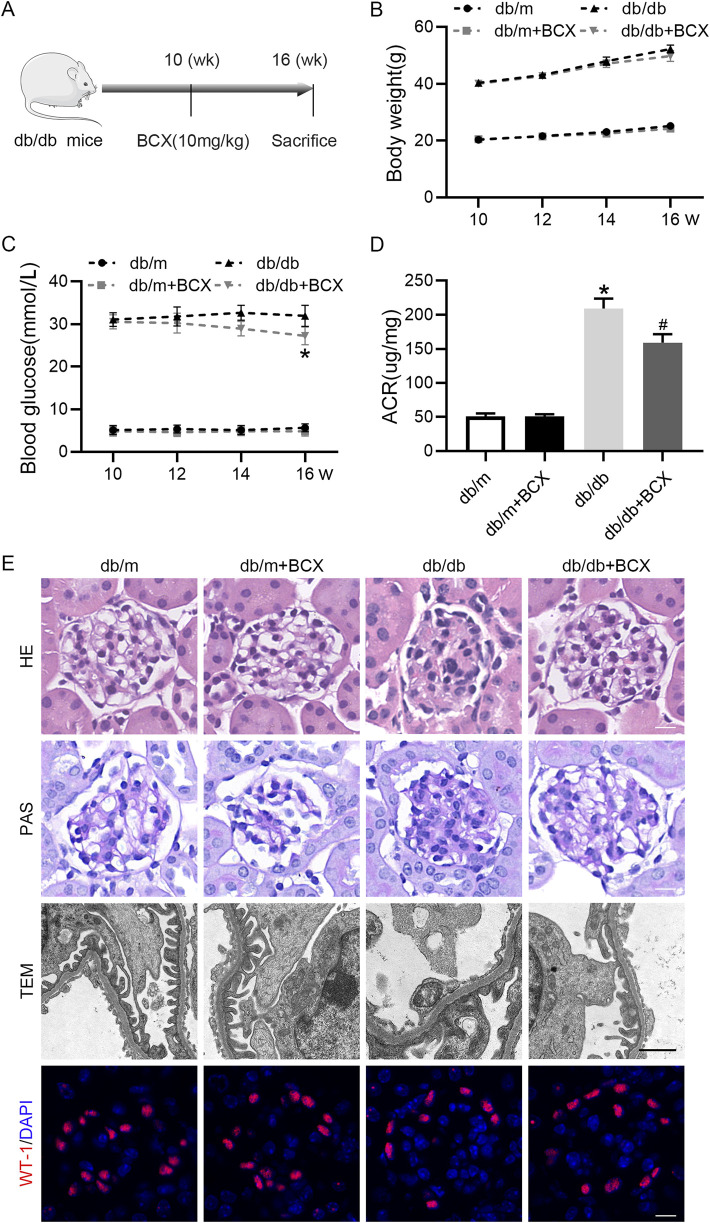
Effects of BCX on functional and morphological characteristics in kidney of db/db mice. **(A)** A schematic diagram showing the intervention-study design. **(B)** Body weight changes in each group. **(C)** Blood glucose levels in each group. **(D)** Urine ACR levels in each group. **(E)** Representative HE stanning images (scale bar = 50 μm), PAS stanning images (scale bar = 50 μm) transmission electron microscopy (TEM) images (scale bar = 1 μm), and Wilms’ Tumor 1 (WT1, red) IF images (scale bar = 50 μm) in glomeruli from each group. *p < 0.05 vs. control group; ^#^ *p* < 0.05 vs. db/db group, n = 6.

First, to evaluate the renal protective effect of BCX, we measured body weight, blood glucose, and urine albumin creatinine ratio (ACR) in mice (Figures 1A–D). The results showed that compared to db/m mice, db/db mice exhibited higher body weight and blood glucose levels. Treatment with BCX alleviated hyperglycemia at 16 weeks but had no effect on body weight (Figures 1B, C). In addition, the ACR level was significantly elevated in db/db mice, and treatment with BCX significantly reduced ACR levels (Figure 1D). Meanwhile, the HE, PAS staining, and TEM were utilized to observe the effect of BCX on the morphological changes of glomeruli in db/db mice (Figure 1E). The results revealed that db/db mice exhibited significant pathological changes, including glomerulosclerosis, mesangial matrix expansion, glomerular basement membrane (GBM) thickening, and diffuse foot process fusion, while the administration of BCX alleviated these pathological changes (Figure 1E). Moreover, WT-1 staining demonstrated that the administration of BCX also reduced diabetes-induced podocyte loss (Figure 1E).

### 3.2 BCX attenuates oxidative stress and aging in glomeruli of db/db mice

**FIGURE 2:**
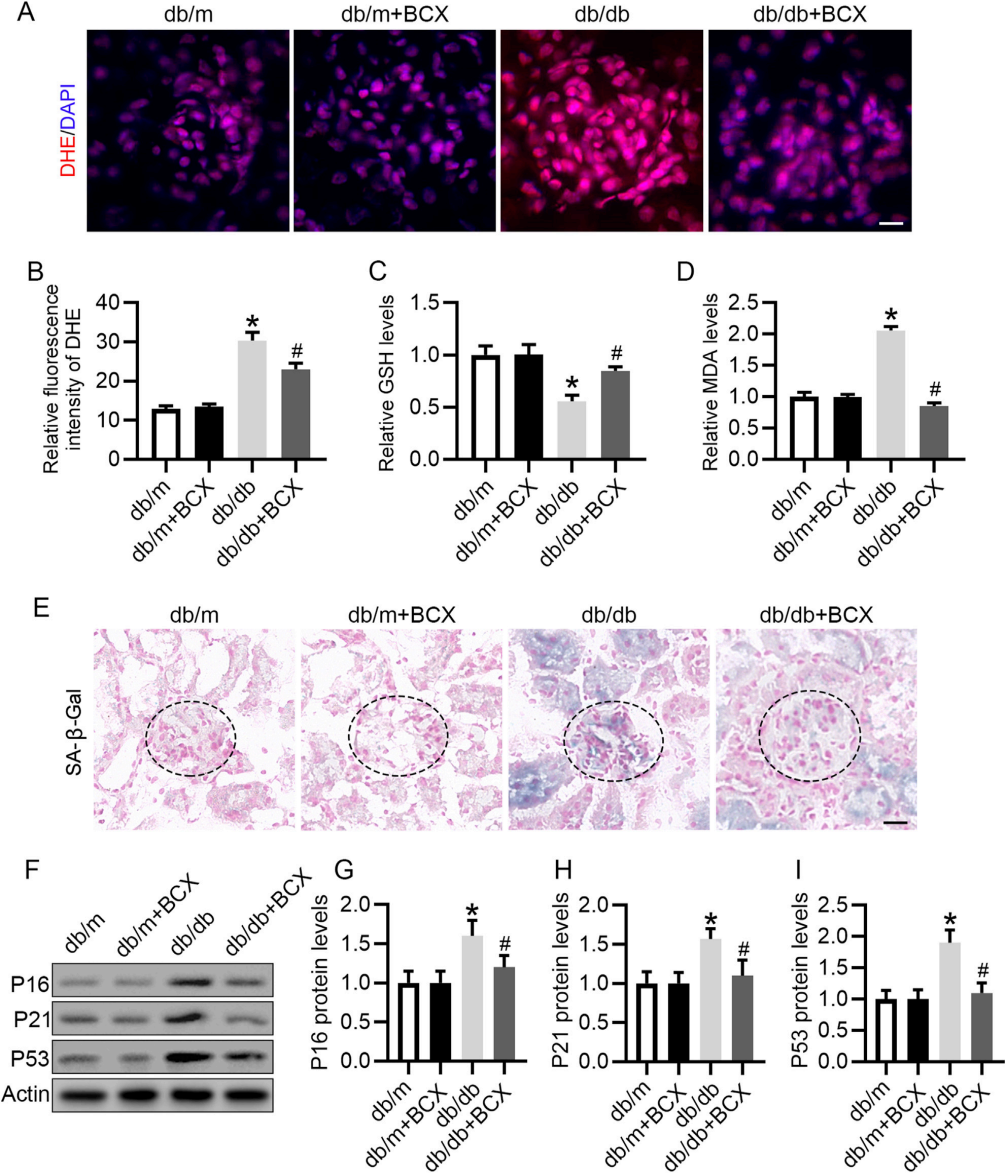
BCX attenuates oxidative stress and aging in glomeruli of db/db mice. **(A)** Oxidative stress level of glomeruli was evaluated by DHE staining from different groups, Scale bar = 10 μm. **(B)** Quantification of DHE fluorescence intensity from panel A. **(C)** GSH concentrations in kidney cortexes from each group. **(D)** MDA concentrations in kidney cortexes from each group. **(E)** Representative SA-β-gal activity staining in kidney from each group, Scale bar = 25 μm. **(F)** Representative images of P16, P21, and P53 protein expression in glomeruli from different groups, n = 6. **(G–I)** Quantitative analysis of P16, P21, and P53 protein levels from panel F *p < 0.05 vs. control group; ^#^ *p* < 0.05 vs. db/db group, n = 6.

Considering the important role oxidative stress plays in glomerular injury in DKD (Su et al., 2023), and since BCX has been shown to exert antioxidant effects (Zhang et al., 2023), we investigated whether BCX could mitigate oxidative stress in glomeruli. DHE staining revealed a significantly increased ROS content in the glomeruli of db/db mice compared with the control group, while treatment with BCX significantly alleviated ROS accumulation in the glomeruli of db/db mice (Figures 2A, B). Additionally, the data showed that GSH level was significantly reduced and MDA level was significantly increased in the renal cortex of db/db mice, while BCX significantly inhibited the reduction of GSH level and the increase of MDA content (Figures 2C, D). Since the accumulation of oxidative stress is closely related to aging, we further examined whether BCX can mitigate kidney aging. The SA-β-gal staining results showed a markedly increased SA-β-gal activity in the glomeruli of db/db mice, which was significantly reduced by BCX treatment (Figure 2E). A defining trait of cellular senescence is cell cycle arrest, which is mainly mediated by p53/p21 and p16 pathways occurring in upregulation of p16, p21 or p53 (Lucas et al., 2023). Interestingly, BCX also inhibited the expression of senescence-related proteins such as P16, P21, and P53 in glomeruli of db/db mice (Figures 2F–I). Collectively, these results indicate that BCX can inhibit oxidative stress damage and aging in the glomeruli of diabetic mice.

### 3.3 BCX ameliorates HG-induced oxidative stress, mitochondrial dysfunction in podocytes

**FIGURE 3:**
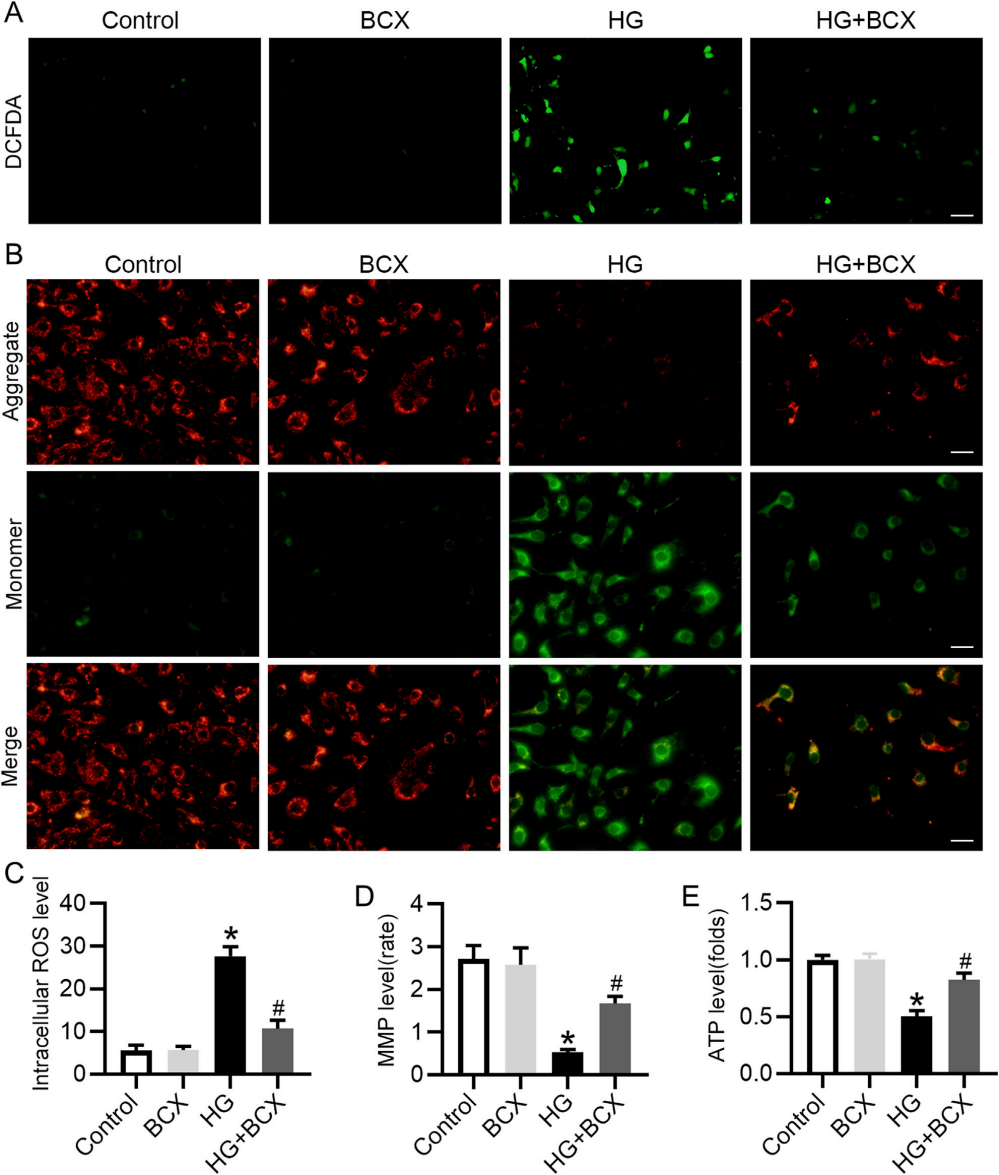
BCX ameliorates HG-induced oxidative stress, mitochondrial dysfunction in podocytes. **(A)** A representative image of DCFDA staining in podocytes from each group, Scale bar = 100 μm, n = 3. **(B)** A representative image of JC-1 staining in podocytes from each group, Scale bar = 100 μm, n = 3. **(C)** Quantitative of ROS content from panel A. **(D)**: Quantitative of mitochondrial membrane potential from panel B. **(E)**: Relative ATP content in podocytes from each group, n = 3. *p < 0.05 vs. control group; ^#^ *p* < 0.05 vs. HG group.

BCX has been shown to attenuate mitochondrial oxidative damage in hepatic cells (Nishino et al., 2021), but its protective effect on podocytes remains unknown. Our study found that HG exposure significantly increased ROS production in podocytes, whereas BCX pre-treatment significantly decreased ROS production (Figures 3A, C). To further investigate the effects of BCX on mitochondrial function, MMP and cellular ATP production were assessed in podocytes. HG exposure significantly decreased MMP and cellular ATP content, while pre-treatment with BCX significantly prevented these changes (Figures 3B–E). Taken together, these results suggest that BCX can mitigate oxidative stress and mitochondrial dysfunction induced by HG in podocytes.

### 3.4 BCX inhibits HG-induced senescence in podocytes

**FIGURE 4:**
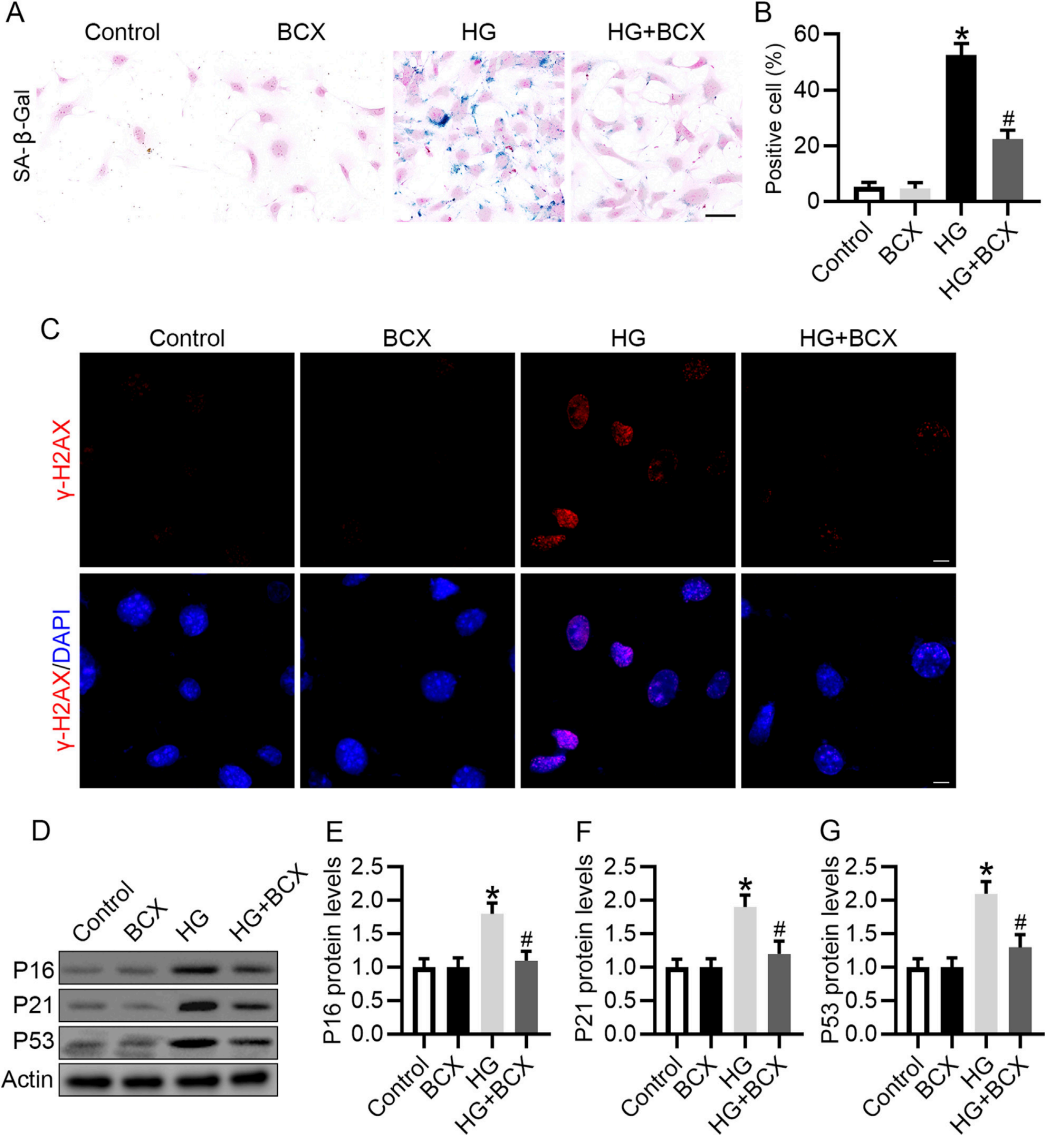
BCX inhibits HG-induced senescence in podocytes. **(A)** Representative SA-β-gal activity staining in podocytes from each group, Scale bar = 50 μm, n = 3. **(B)** Quantification of positive cells from panel A. **(C)** Representative γ-H2AX staining (red) in podocytes from each group, Scale bar = 10 μm, n = 3. **(D)** Representative images of P16, P21, and P53 protein expression in podocytes from different groups, n = 3. **(E–G)** Quantitative analysis of P16, P21, and P53 protein levels from panel D. *p < 0.05 vs. control group; ^#^ *p* < 0.05 vs. HG group.

*In vivo* studies have confirmed that BCX can reduce kidney aging in db/db mice. We further explored whether BCX can reduce HG-induced podocyte senescence *in vitro*. SA-β-gal staining showed that HG significantly increase the proportion of SA-β-gal-positive cells, and pre-treatment with BCX significantly reduced SA-β-gal-positive cells (Figures 4A, B). In addition, γ-H2AX (a cellular senescence marker) staining showed that BCX markedly decreased the expression of γ-H2AX in podocytes induced by HG (Figure 4C). Moreover, BCX also inhibited HG-induced increases in senescence-related proteins such as P16, P21, and P53 (Figures 4D–G). Together, these results suggest that BCX alleviates HG-induced podocyte senescence *in vitro*.

### 3.5 Effects of BCX on Nrf2/HO-1 pathway in podocytes both *in vivo* and *in vitro*

**FIGURE 5:**
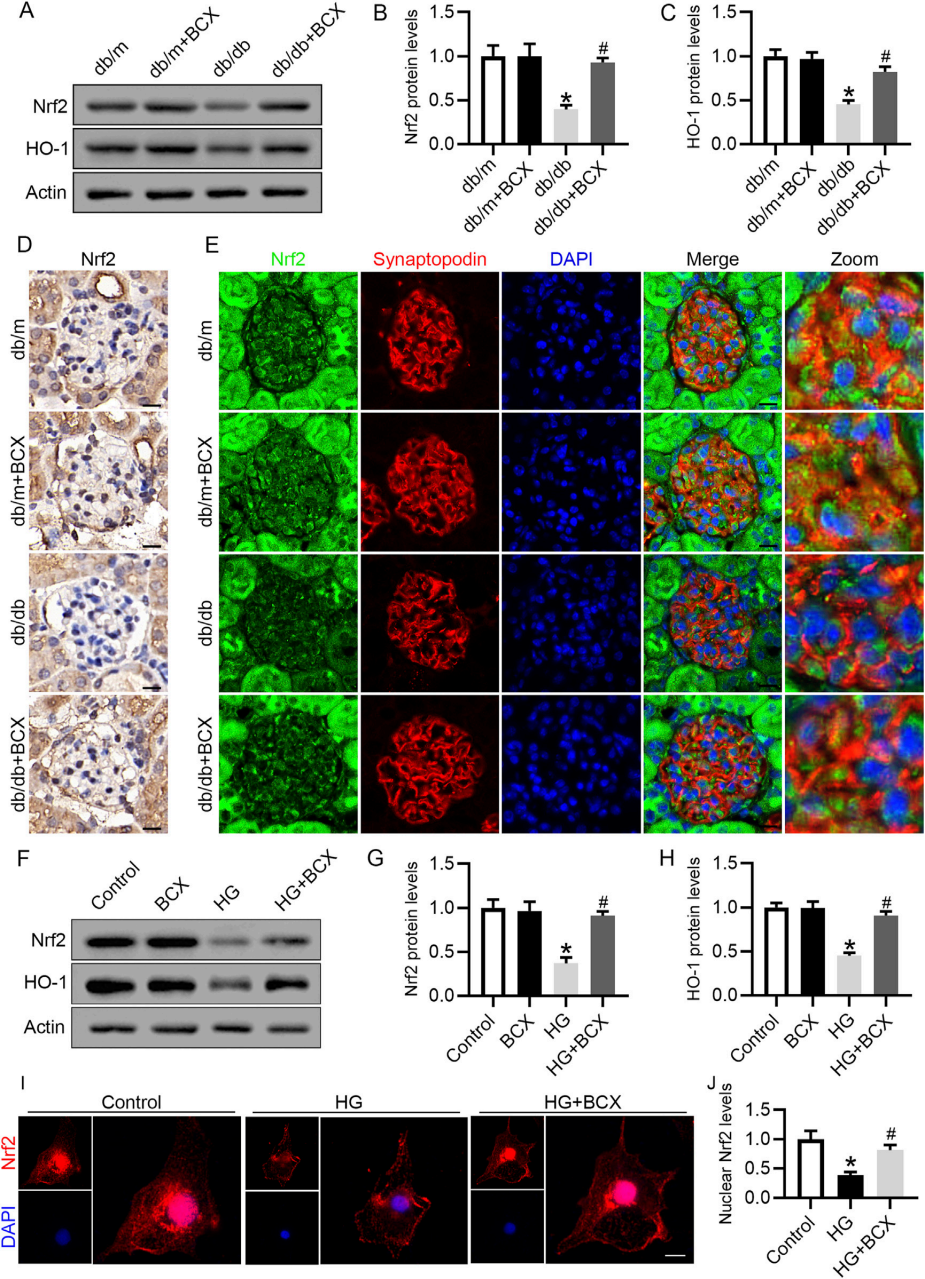
Effects of BCX on Nrf2/HO-1 pathway in podocytes *in vivo* and *in vitro* **(A)** Representative images of Nrf2 and HO-1 protein expression in glomeruli in the different groups, n = 6. **(B, C)** Quantitative analysis of Nrf2 and HO-1 protein levels from panel A. **(D)** Immunohistochemical analysis of Nrf2 in glomeruli from different groups, Scale bar = 10 μm, n = 6. **(E)** Representative fluorescence microscope images of Nrf2 (green), Synaptopodin (red), DAPI (blue) in glomeruli from different groups, Scale bar = 10 μm, n = 6. **(F)** Representative images of Nrf2 and HO-1 protein expression in podocytes, n = 3. **(G, H)** Quantitative analysis of Nrf2 and HO-1 protein levels from panel F. **(I)** Representative fluorescence microscope images of Nrf2 in podocytes, Scale bar = 20 μm, n = 3. **(J)** Quantitative analysis of nuclear expression levels of Nrf2. *p < 0.05 vs. db/m or control group; ^#^ *p* < 0.05 vs. db/db or HG group.

Based on the previous results, we speculated that the protective effect of BCX against oxidative stress, mitochondrial dysfunction and senescence might be attributed to the activation of antioxidant genes. Since Nrf2/heme oxygenase-1 (HO-1) signaling pathway plays a key role in antioxidative stress (Lin et al., 2023), we investigated whether Nrf2 activation was involved in the protective effects of BCX. Western blot analysis revealed a significant decrease in Nrf2 and HO-1 protein levels in the glomeruli of db/db mice compared to db/m mice (Figures 5A–C). Moreover, the reduced Nrf2 protein expression in glomeruli and podocytes from db/db mice were also confirmed by immunohistochemical and immunofluorescence double staining (Figures 5D, E). Interestingly, BCX treatment reversed the reduction of Nrf2 and HO-1 protein levels in glomeruli and podocytes from db/db mice (Figures 5D, E). Similar to the results observed *in vivo*, the Nrf2 and HO-1 expression were decreased in HG-stimulated podocytes, which was also reversed by BCX pre-treatment (Figures 5F–H). Furthermore, immunofluorescence was used to monitor the nuclear translocation of Nrf2. The results revealed a significant decrease in nuclear Nrf2 expression in HG-stimulated podocytes, which was restored by BCX pre-treatment (Figures 5I, J). Taken together, these data suggest that Nrf2 signaling may be involved in the protective effects of BCX against HG-induced oxidative stress, mitochondrial dysfunction, and senescence in podocytes.

### 3.6 Knockdown of Nrf2 blocked anti-oxidative stress effect of BCX in podocytes

**FIGURE 6:**
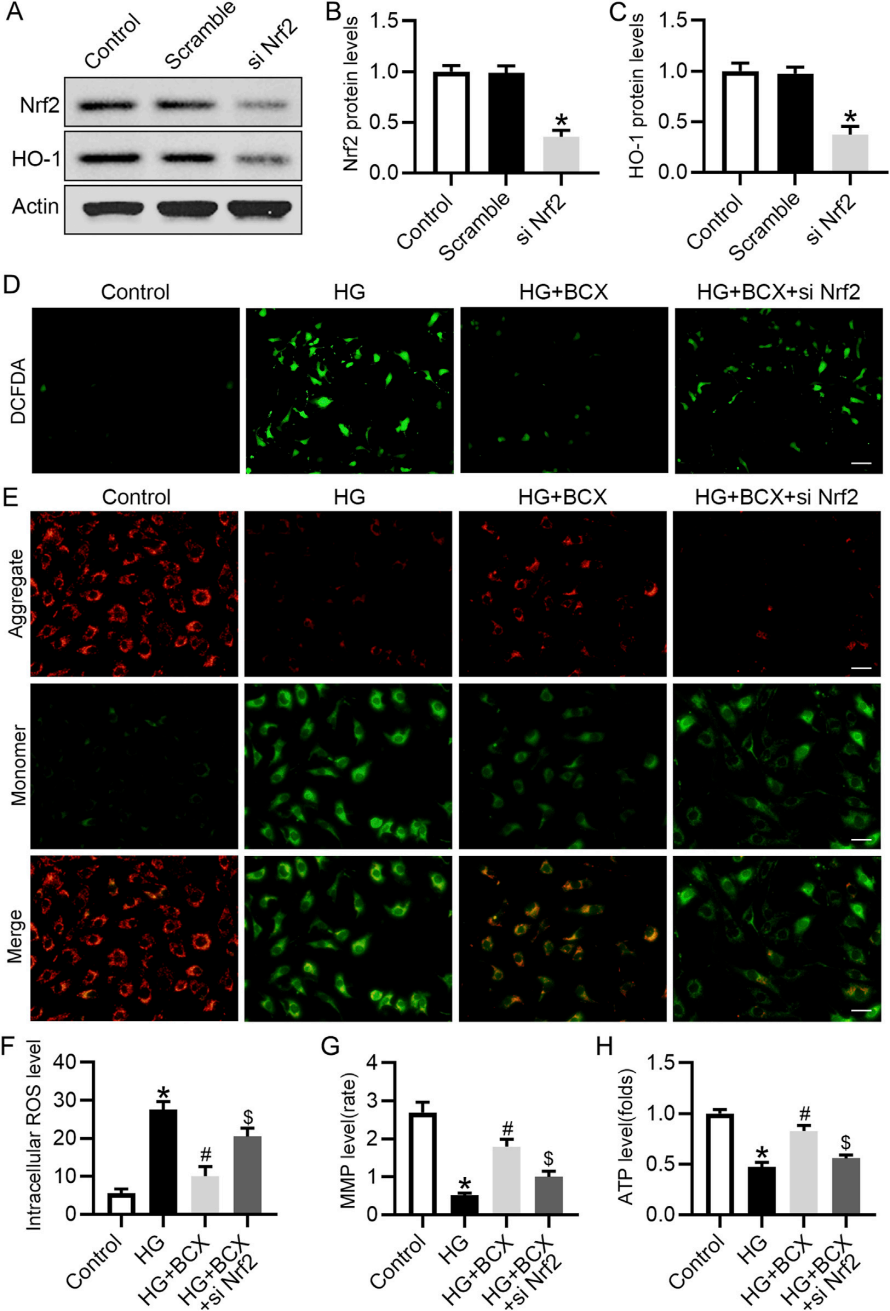
Knockdown of Nrf2 blocked anti-oxidative stress effect of BCX in podocytes. **(A)** Representative images of Nrf2 and HO-1 protein expression in podocytes, n = 3. **(B, C)** Quantitative analysis of Nrf2 and HO-1 protein levels from panel A. **(D)** A representative image of DCFDA staining in podocytes from each group, Scale bar = 100 μm, n = 3. **(E)** A representative image of JC-1 staining in podocytes from each group, Scale bar = 100 μm, n = 3. **(F)** Quantitative of ROS content from panel D. **(G)**: Quantitative of mitochondrial membrane potential from panel E. **(H)**: Relative ATP content in podocytes from each group, n = 3. *p < 0.05 vs. control group; ^#^ *p* < 0.05 vs. HG group, ^$^ *p* < 0.05 vs. HG + BCX group.

To further validate the role of Nrf2 in the mitigation of oxidative stress and protection of mitochondrial function by BCX, Nrf2 siRNA was used to knockdown the expression of Nrf2 (Figures 6A–C). DCFH-DA staining showed that the ROS content was significantly increased in podocytes under HG conditions, but was decreased by BCX pre-treatment, and this effect was partially abolished by Nrf2 siRNA transfection (Figures 6D, F). In addition, BCX inhibited the HG-induced decrease in MMP and ATP levels, and these effects were abolished by Nrf2 siRNA transfection (Figures 6E–H). These results suggest that BCX mitigates oxidative stress and mitochondrial damage partially through the Nrf2/HO-1 pathway.

### 3.7 Knockdown of Nrf2 blocked the anti-senescence effect of BCX in podocytes

**FIGURE 7:**
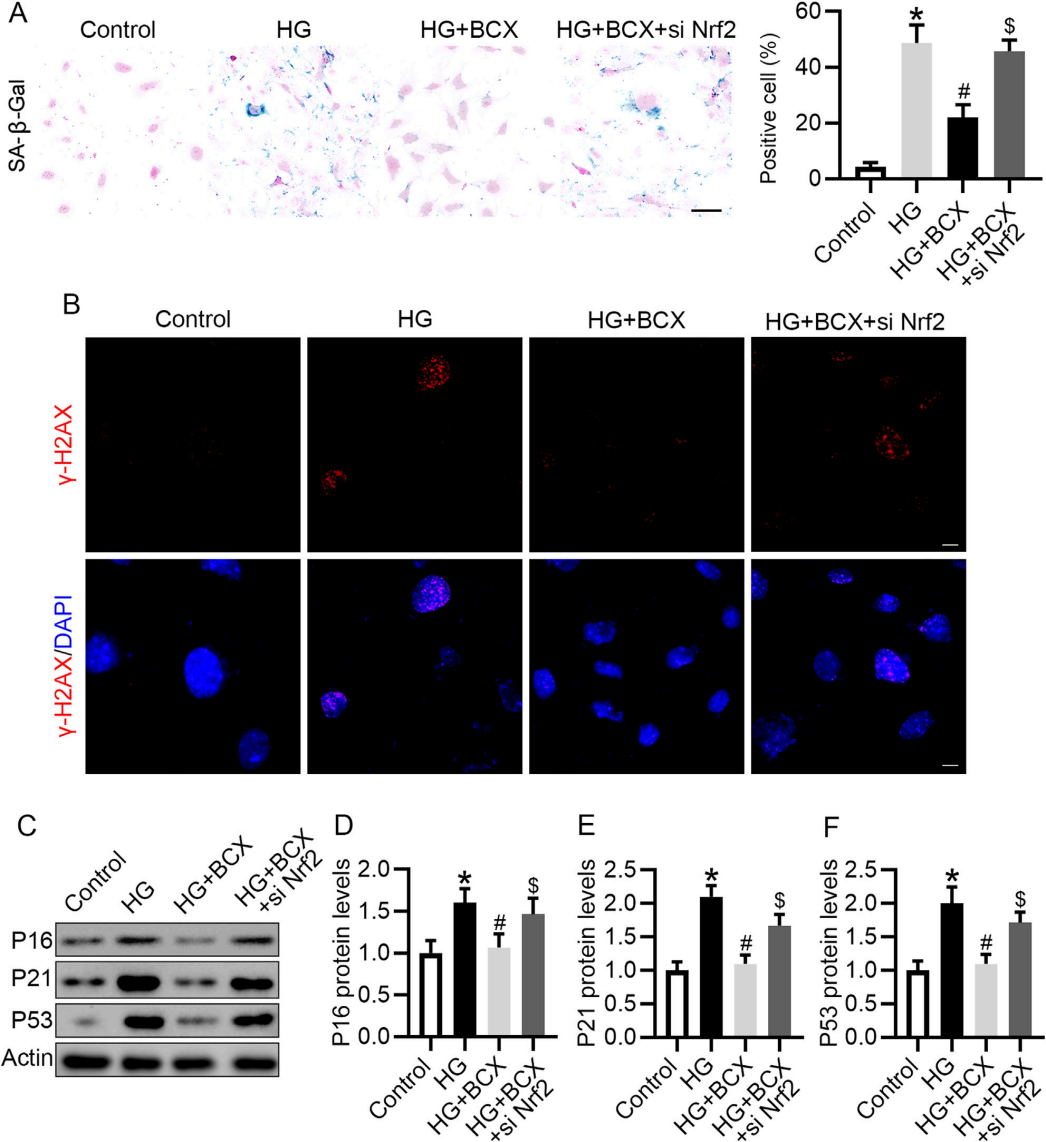
Knockdown of Nrf2 blocked the anti-senescence effect of BCX in podocytes. **(A)** Representative SA-β-gal activity staining in podocytes and the quantification of positive cells from each group, Scale bar = 50 μm, n = 3. **(B)** Representative γ-H2AX staining (red) in podocytes from each group, Scale bar = 10 μm, n = 3. **(C)** Representative images of P16, P21, and P53 protein expression in podocytes from different groups, n = 3. **(D–F)** Quantitative analysis of P16, P21, and P53 protein levels from **(D)**. *p < 0.05 vs. control group; ^#^ *p* < 0.05 vs. HG group, ^$^ *p* < 0.05 vs. HG + BCX group.

Next, we evaluated whether BCX plays an anti-senescence role by activating Nrf2. BCX significantly reduced the number of senescent cells under HG conditions, however, knockdown of Nrf2 partially abolished the effect of BCX on the clearance of senescent cells (Figure 7A). Consistent with previous results, pre-treatment with BCX significantly decreased the expression of senescence-related proteins, including γ-H2AX, P16, P21, and P53, while knockdown of Nrf2 blocked the downregulation of senescence-related proteins induced by BCX treatment (Figures 7B–F).

### 3.8 Silencing Nrf2 reversed anti-apoptotic effects of BCX in podocytes

**FIGURE 8:**
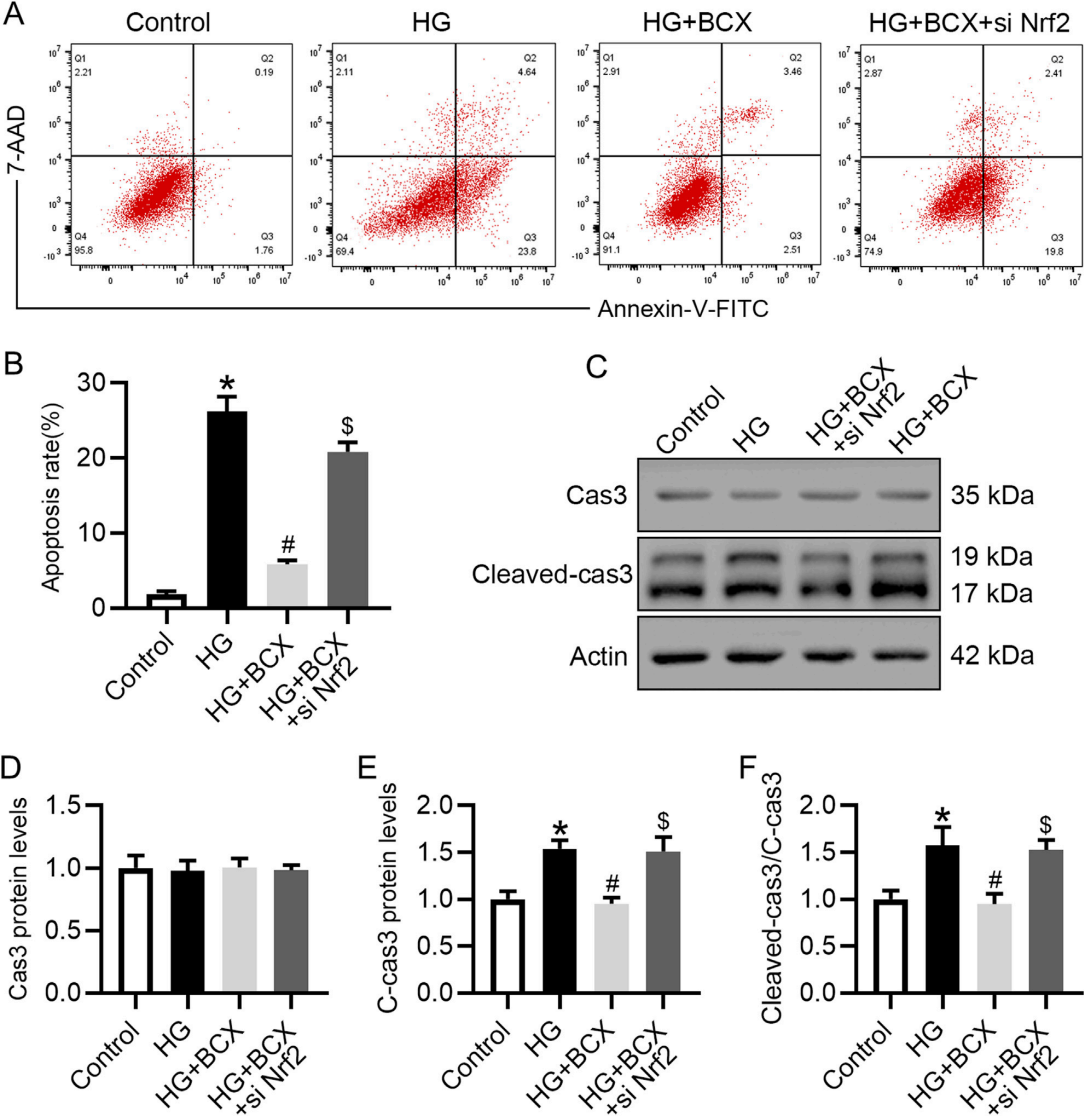
Silencing Nrf2 reversed the protective effect of BCX against the HG-induced podocyte apoptosis. **(A)** Flow cytometry analysis of podocyte apoptosis in different groups, n = 3. **(B)** The apoptosis rates from panel A. **(C)** Representative images of Cas3 and cleaved-cas3 protein expression in podocytes, n = 3. **(D–F)** Quantitative analysis of Cas3, cleaved-cas3 protein levels and cleaved-cas3/Cas3 ration from panel C. *p < 0.05 vs. control group; ^#^ *p* < 0.05 vs. HG group, ^$^ *p* < 0.05 vs. HG + BCX group.

Previous studies have shown that oxidative stress, mitochondrial damage and senescence can initiate cellular apoptosis (Su et al., 2023). Therefore, we also explored whether BCX could ameliorate podocyte apoptosis under HG conditions and evaluated the role of Nrf2 in this process. Podocyte apoptosis was measured using flow cytometry, and the data indicated that treatment with BCX significantly ameliorated HG-induced podocyte apoptosis. However, this effect was partially diminished by Nrf2 siRNA transfection (Figures 8A, B). In addition, the Western blot showed that HG increased the expression of apoptosis marker cleaved-caspase3 (cleaved-cas3) rather than total Cas3 in podocytes, and BCX treatment decreased upregulated cleaved-cas3, and this effect was eliminated by Nrf2 siRNA transfection (Figures 8C–F).

## 4 Discussion

**FIGURE 9:**
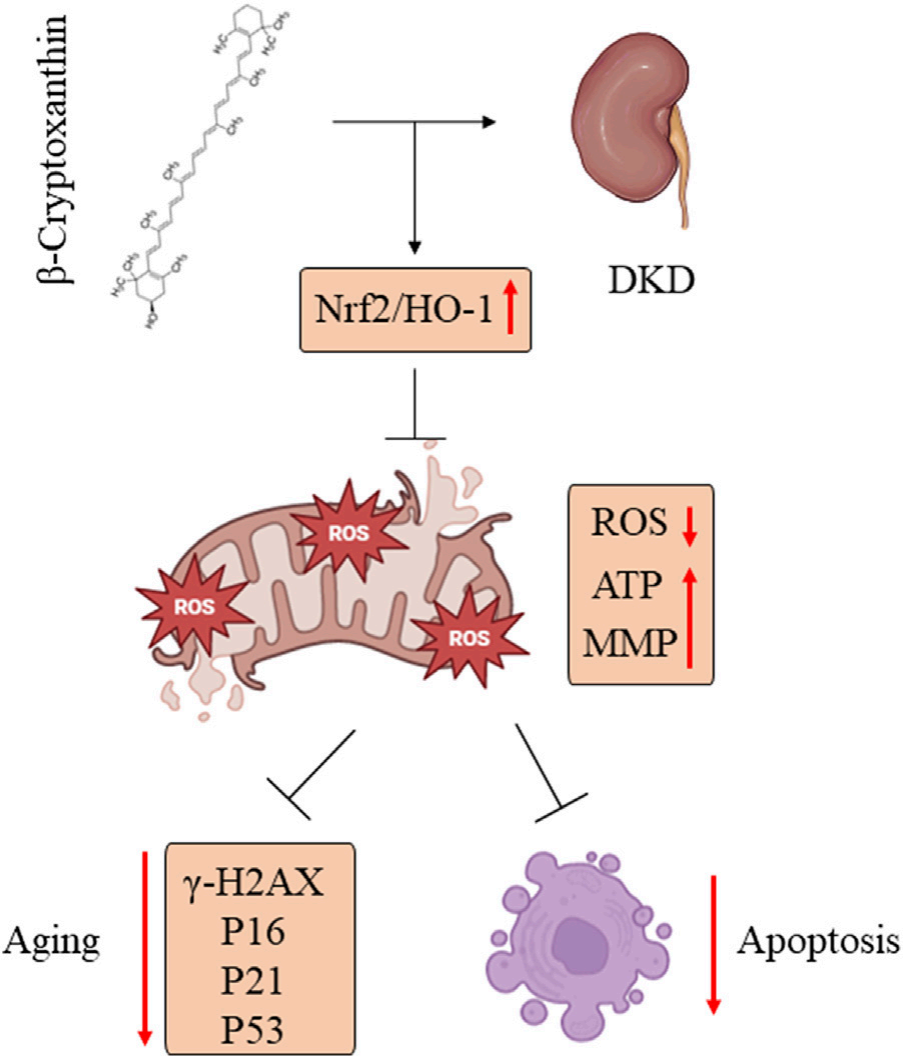
BCX alleviated podocyte oxidative stress, mitochondrial dysfunction, and senescence in DKD by promoting Nrf2/HO-1 signaling pathways.

In the present study, we demonstrated that BCX was able to inhibit HG-induced oxidative stress, mitochondrial damage, and senescence in podocytes. Additionally, we explored the potential mechanisms underlying these effects. Our findings indicated that the protective effect of BCX on podocytes is partly mediated by activating the Nrf2/HO-1 signaling pathway (Figure 9). Overall, our findings provide experimental support for further investigation of the protective role of BCX in DKD.

Numerous studies have established that oxidative stress is a major factor in the development and progression of DKD (Ma et al., 2023; Wu et al., 2023a). The kidney is one of the most energy-demanding organs and is second only to the heart in the expression of proteins related to mitochondrial function and oxygen consumption (Hoogstraten et al., 2024). This enormous energy consumption can sometimes lead to increased oxidative stress (Teixeira et al., 2023). In the oxidative phosphorylation (OXPHOS) pathway, the electron transport chain (ETC) generates most of the mitochondrial ROS (mtDNA), and excessive ROS production progressively damages mitochondria, leading to a decrease in the efficiency of the ETS, a further increase in the level of ROS and a decrease in the MMP and ATP production, which ultimately trigger apoptosis and senescence (Raut and Khullar, 2023). Furthermore, oxidative stress is a crucial factor driving senescence of podocytes (Chen et al., 2024). ROS can cause DNA damage and double-strand breaks, activating the p53-mediated DNA damage response, which upregulates p21 and p16, leading to the cell cycle arrest and checkpoint enforcement, ultimately resulting in cellular senescence (Achanta and Huang, 2004). Recent studies have also shown that oxidative damage and senescence of podocytes are involved in the progression of DKD (Chen et al., 2024). Our study confirmed that HG can induce oxidative stress, mitochondrial damage, and apoptosis in podocytes. Besides, our data showed that HG stimulation increases the number of SA-β-gal positive cells, as well as the expression of P16, P21 and P53, and promotes the production of the cellular senescence marker γ-H2AX in podocyte. Therefore, inhibiting oxidative stress damage and senescence is an important measure to protect podocyte injury in the diabetic state.

Currently, many researchers are focusing on the development of potent antioxidant drugs (Sakashita et al., 2021; Wu et al., 2023b), such as MitoQ and SS-31 (Kirkman et al., 2023; Shan et al., 2023). These antioxidants have shown efficacy in treating kidney diseases (Hou et al., 2016; Xiao et al., 2017). For example, MitoQ has been demonstrated to attenuate renal tubular oxidative stress injury by reducing mitochondrial fission and promoting mitochondrial autophagy (Xiao et al., 2017). SS-31 is a mitochondria-targeted tetrapeptide that can scavenge mtROS and inhibit the opening of the mitochondrial permeability transition pore (mPTP) (Szeto et al., 2011). SS-31 significantly reduces oxidative stress and inflammation after ischemia-reperfusion (IR) injury and promote the proliferation of surviving renal tubular cells (Szeto et al., 2011). The clinical potential of such synthetic antioxidants has been described but has not yet advanced to the clinical trial stage (Gutierrez-Mariscal et al., 2020). In addition, the cost of synthetic antioxidants is relatively high, making them unsuitable for widespread use. Therefore, the search for natural antioxidants with minimal side effects and lower costs, suitable for use in foods or as medicine, remains an active area of research.

Humans primarily obtain dietary carotenoids from foods such as green and yellow vegetables and algae (Wang et al., 2024). Carotenoids have been reported to be effective in reducing the risk of lifestyle-related diseases (Terao, 2023). BCX is an antioxidant carotenoid that has recently gained attention for its role in reducing the risk of NAFLD (Clugston, 2023). BCX ameliorates NAFLD progression through a multifaceted approach, primarily due to its antioxidative stress effects (Clugston, 2023). A recent study has also found that BCX attenuates the development of NAFLD in mice by suppressing inflammation (Haidari et al., 2020). In addition, BCX has been shown to prevent cognitive dysfunction and oxidative damage in aging mice (Ma et al., 2023). This evidence suggests that BCX is an antioxidant with therapeutic potential; however, its efficacy in delaying the progression of DKD remains unclear. We have demonstrated for the first time that BCX lowers blood glucose and attenuates glomerulosclerosis and proteinuria levels in diabetic mice. Furthermore, our findings indicate that BCX inhibited HG-induced oxidative stress, mitochondrial damage, senescence and apoptosis in podocytes *in vitro*.

The present study also explored the underlying mechanism by which BCX regulates oxidative stress, mitochondrial damage in podocytes. It is well-known that Nrf2 is a key transcription factor for maintaining intracellular redox homeostasis, and delaying cellular senescence (Lin et al., 2023). Under physiological conditions, Nrf2 is retained in the cytoplasm by Kelch-like ECH-associated protein 1 (Keap1) and is degraded through ubiquitination (Dinkova-Kostova and Copple, 2023). However, upon oxidative stimulation or in the presence of Nrf2-stimulating factors, Nrf2 dissociates from Keap1 and then translocate to the nucleus, where it activates a series of cell-protective and antioxidant genes, including HO-1 and NAD(P)H quinone dehydrogenase 1(NQO-1) (Dinkova-Kostova and Copple, 2023). Numerous studies have confirmed that Nrf2/HO-1 pathway has a protective effect on renal oxidative stress injury in diabetic mice (Liu et al., 2022b; Xiao et al., 2017). Activation of Nrf2 can delay the progression of DKD by inhibiting mitochondrial fission, ferroptosis, and inflammatory responses as well as promoting mitochondrial autophagy (Lu et al., 2023; Xiao et al., 2017). However, it remains uncertain whether BCX can attenuate HG-induced podocyte injury by activating Nrf2. A recent study found that BCX maintained mitochondrial function by promoting Nrf2 nuclear translocation to inhibit oxidative stress in HK-2 cells (Zhang et al., 2023). To determine whether BCX also activates Nrf2 in podocytes, we assessed the expression of Nrf2 and HO-1 by different methods. Our results showed that BCX reversed HG-induced downregulation of Nrf2 and HO-1 in podocytes both *in vivo* and *in vitro*. Furthermore, BCX promoted Nrf2 nuclear translocation to enhance its anti-oxidative stress capacity. Importantly, knockdown of Nrf2 in podocytes partially abolished the anti-oxidative stress and mitochondrial protective effects of BCX, suggesting that BCX confers its protective effects by activating Nrf2/HO-1 signaling pathway.

Currently, the research on BCX primarily focuses on fundamental mechanism studies and lacks convincing clinical research, particularly large-scale prospective randomized controlled trials (RCTs). The most notable study is the ‘Mikkabi Cohort Study’ conducted in Japan, which initially investigated the association between serum concentrations of six dietary carotenoids (including BCX) and the risk of developing lifestyle-related diseases among residents in Mikkabi town, Shizuoka Prefecture. This study revealed a negative correlation between BCX and the incidence of dyslipidemia and type 2 diabetes mellitus; however, it did not address potential adverse reactions associated with BCX (Sugiura et al., 2015a; Sugiura et al., 2015b). Therefore, there is a need for further investigation into potential adverse reactions that may arise from extensive clinical application of BCX as a pharmaceutical agent - an issue that should be addressed by the scientific community. Nevertheless, based on these findings and other research conclusions, we believe that BCX exhibits promising potential for the treatment of DKD and warrants further exploration through RCT studies.

This study also has some limitations. First, our research did not reveal how BCX activates the Nrf2 pathway. Glycogen synthase kinase 3β (GSK) may be the upstream molecule regulating Nrf2, which recent studies have confirmed that GSK3β is hyperactive in glomerular podocytes, associated with impaired Nrf2 response and premature senescence (Chen et al., 2024). Second, although this study confirmed that BCX can activate Nrf2, it did not verify *in vivo* experiments whether inhibiting Nrf2 could block the protective effect of BCX on podocyte injury in db/db mice.

In conclusion, this study demonstrates for the first time that BCX, as an antioxidant, prevents hyperglycemic-induced podocyte injury by inhibiting oxidative stress and mitochondrial dysfunction by activating the Nrf2/HO-1 signaling pathway. These findings strongly support a therapeutic potential of BCX in the treatment of podocyte injury under diabetic conditions.

## Data Availability

The original contributions presented in the study are included in the article/supplementary material, further inquiries can be directed to the corresponding authors.

## References

[B1] Achanta G. Huang P. (2004). Role of p53 in sensing oxidative DNA damage in response to reactive oxygen species-generating agents. Cancer Res. 64, 6233–6239. 10.1158/0008-5472.CAN-04-0494 15342409

[B2] Chae S. Y. Kim Y. Park C. W. (2023). Oxidative stress induced by lipotoxicity and renal hypoxia in diabetic kidney disease and possible therapeutic interventions: targeting the lipid metabolism and hypoxia. Antioxidants-Basel 12, 2083. 10.3390/antiox12122083 38136203 PMC10740440

[B3] Chen M. Fang Y. Ge Y. Qiu S. Dworkin L. Gong R. (2024). The redox-sensitive GSK3β is a key regulator of glomerular podocyte injury in type 2 diabetic kidney disease. Redox Biol. 72, 103127. 10.1016/j.redox.2024.103127 38527400 PMC10979123

[B4] Clugston R. D. (2023). β-cryptoxanthin and fatty liver disease: new insights. Hepatobil Surg. Nutr. 12, 450–452. 10.21037/hbsn-23-201 PMC10282678 37351127

[B5] Dai Z. C. Chen J. X. Zou R. Liang X. B. Tang J. X. Yao C. W. (2023). Role and mechanisms of SGLT-2 inhibitors in the treatment of diabetic kidney disease. Front. Immunol. 14, 1213473. 10.3389/fimmu.2023.1213473 37809091 PMC10552262

[B6] Dinkova-Kostova A. T. Copple I. M. (2023). Advances and challenges in therapeutic targeting of NRF2. Trends Pharmacol. Sci. 44, 137–149. 10.1016/j.tips.2022.12.003 36628798

[B7] Fu Y. Sun Y. Wang M. Hou Y. Huang W. Zhou D. (2020). Elevation of JAML promotes diabetic kidney disease by modulating podocyte lipid metabolism. Cell Metab. 32, 1052–1062. 10.1016/j.cmet.2020.10.019 33186558

[B8] Granot-Hershkovitz E. He S. Bressler J. Yu B. Tarraf W. Rebholz C. M. (2023). Plasma metabolites associated with cognitive function across race/ethnicities affirming the importance of healthy nutrition. Alzheimers Dement. 19, 1331–1342. 10.1002/alz.12786 36111689 PMC10017373

[B9] Gutierrez-Mariscal F. M. Arenas-de L. A. Limia-Perez L. Romero-Cabrera J. L. Yubero-Serrano E. M. López-Miranda J. (2020). Coenzyme Q (10) supplementation for the reduction of oxidative stress: clinical implications in the treatment of chronic diseases. Int. J. Mol. Sci. 21, 7870. 10.3390/ijms21217870 33114148 PMC7660335

[B10] Haidari F. Hojhabrimanesh A. Helli B. Seyedian S. S. Ahmadi-Angali K. (2020). An energy-restricted high-protein diet supplemented with β-cryptoxanthin alleviated oxidative stress and inflammation in nonalcoholic fatty liver disease: a randomized controlled trial. Nutr. Res. 73, 15–26. 10.1016/j.nutres.2019.08.009 31841744

[B11] Hoogstraten C. A. Hoenderop J. G. de Baaij J. (2024). Mitochondrial dysfunction in kidney tubulopathies. Annu. Rev. Physiol. 86, 379–403. 10.1146/annurev-physiol-042222-025000 38012047

[B12] Hou Y. Li S. Wu M. Wei J. Ren Y. Du C. (2016). Mitochondria-targeted peptide SS-31 attenuates renal injury via an antioxidant effect in diabetic nephropathy. Am. J. Physiol-Renal 310, F547–F559. 10.1152/ajprenal.00574.2014 26719366

[B13] Jomova K. Raptova R. Alomar S. Y. Alwasel S. H. Nepovimova E. Kuca K. (2023). Reactive oxygen species, toxicity, oxidative stress, and antioxidants: chronic diseases and aging. Arch. Toxicol. 97, 2499–2574. 10.1007/s00204-023-03562-9 37597078 PMC10475008

[B14] Kirkman D. L. Stock J. M. Shenouda N. Bohmke N. J. Kim Y. Kidd J. (2023). Effects of a mitochondrial-targeted ubiquinol on vascular function and exercise capacity in chronic kidney disease: a randomized controlled pilot study. Am. J. Physiol-Renal 325, F448–F456. 10.1152/ajprenal.00067.2023 37560769

[B15] Li D. Yu Q. Wu R. Tuo Z. Wang J. Ye L. (2024). Interactions between oxidative stress and senescence in cancer: mechanisms, therapeutic implications, and future perspectives. Redox Biol. 73, 103208. 10.1016/j.redox.2024.103208 38851002 PMC11201350

[B16] Li X. Zhang Y. Xing X. Li M. Liu Y. Xu A. (2023). Podocyte injury of diabetic nephropathy: novel mechanism discovery and therapeutic prospects. Biomed. Pharmacother. 168, 115670. 10.1016/j.biopha.2023.115670 37837883

[B17] Lin D. W. Hsu Y. C. Chang C. C. Hsieh C. C. Lin C. L. (2023). Insights into the molecular mechanisms of NRF2 in kidney injury and diseases. Int. J. Mol. Sci. 24, 6053. 10.3390/ijms24076053 37047024 PMC10094034

[B18] Liu C. Rafacho B. Wang X. D. (2022a). Xanthophyll β-cryptoxanthin treatment inhibits hepatic steatosis without altering vitamin A status in β-carotene 9',10'-oxygenase knockout mice. Hepatobil Surg. Nutr. 11, 188–198. 10.21037/hbsn-20-404 PMC9023830 35464265

[B19] Liu Y. Uruno A. Saito R. Matsukawa N. Hishinuma E. Saigusa D. (2022b). Nrf2 deficiency deteriorates diabetic kidney disease in Akita model mice. Redox Biol. 58, 102525. 10.1016/j.redox.2022.102525 36335764 PMC9641024

[B20] Liu Y. Wang S. Jin G. Gao K. Wang S. Zhang X. (2023). Network pharmacology-based study on the mechanism of ShenKang injection in diabetic kidney disease through Keap1/Nrf2/Ho-1 signaling pathway. Phytomedicine 118, 154915. 10.1016/j.phymed.2023.154915 37392674

[B21] Lizotte F. Rousseau M. Denhez B. Lévesque D. Guay A. Liu H. (2023). Deletion of protein tyrosine phosphatase SHP-1 restores SUMOylation of podocin and reverses the progression of diabetic kidney disease. Kidney Int. 104, 787–802. 10.1016/j.kint.2023.06.038 37507049

[B22] Lu Q. Yang L. Xiao J. J. Liu Q. Ni L. Hu J. W. (2023). Empagliflozin attenuates the renal tubular ferroptosis in diabetic kidney disease through AMPK/NRF2 pathway. Free Radic. Bio Med. 195, 89–102. 10.1016/j.freeradbiomed.2022.12.088 36581059

[B23] Lucas V. Cavadas C. Aveleira C. A. (2023). Cellular senescence: from mechanisms to current biomarkers and senotherapies. Pharmacol. Rev. 75, 675–713. 10.1124/pharmrev.122.000622 36732079

[B24] Ma X. Ma J. Leng T. Yuan Z. Hu T. Liu Q. (2023). Advances in oxidative stress in pathogenesis of diabetic kidney disease and efficacy of TCM intervention. Ren. Fail. 45, 2146512. 10.1080/0886022X.2022.2146512 36762989 PMC9930779

[B25] Mohandes S. Doke T. Hu H. Mukhi D. Dhillon P. Susztak K. (2023). Molecular pathways that drive diabetic kidney disease. J. Clin. Invest. 133, e165654. 10.1172/JCI165654 36787250 PMC9927939

[B26] Nishino A. Maoka T. Yasui H. (2021). Preventive effects of β-cryptoxanthin, a potent antioxidant and provitamin A carotenoid, on lifestyle-related diseases-A central focus on its effects on non-alcoholic fatty liver disease (NAFLD). Antioxidants-Basel 11, 43. 10.3390/antiox11010043 35052547 PMC8772992

[B27] Raut S. K. Khullar M. (2023). Oxidative stress in metabolic diseases: current scenario and therapeutic relevance. Mol. CELL Biochem. 478, 185–196. 10.1007/s11010-022-04496-z 35764861

[B28] Rayego-Mateos S. Rodrigues-Diez R. R. Fernandez-Fernandez B. Mora-Fernández C. Marchant V. Donate-Correa J. (2023). Targeting inflammation to treat diabetic kidney disease: the road to 2030. Kidney Int. 103, 282–296. 10.1016/j.kint.2022.10.030 36470394

[B29] Sakashita M. Tanaka T. Inagi R. (2021). Metabolic changes and oxidative stress in diabetic kidney disease. Antioxidants-Basel 10, 1143. 10.3390/antiox10071143 34356375 PMC8301131

[B30] Salemkour Y. Yildiz D. Dionet L. T H. D. Verheijden K. Saito R. (2023). Podocyte injury in diabetic kidney disease in mouse models involves TRPC6-mediated calpain activation impairing autophagy. J. Am. Soc. Nephrol. 34, 1823–1842. 10.1681/ASN.0000000000000212 37678257 PMC10631601

[B31] Shan Z. Wang Y. Qiu T. Zhou Y. Zhang Y. Hu L. (2023). SS-31 alleviated nociceptive responses and restored mitochondrial function in a headache mouse model via Sirt3/Pgc-1α positive feedback loop. J. Headache Pain 24, 65. 10.1186/s10194-023-01600-6 37271805 PMC10240765

[B32] Su S. Ma Z. Wu H. Xu Z. Yi H. (2023). Oxidative stress as a culprit in diabetic kidney disease. Life Sci. 322, 121661. 10.1016/j.lfs.2023.121661 37028547

[B33] Sugiura M. Nakamura M. Ogawa K. Ikoma Y. Yano M. (2015a). High serum carotenoids associated with lower risk for the metabolic syndrome and its components among Japanese subjects: Mikkabi cohort study. Brit J. Nutr. 114, 1674–1682. 10.1017/S0007114515003268 26365147

[B34] Sugiura M. Nakamura M. Ogawa K. Ikoma Y. Yano M. (2015b). High-serum carotenoids associated with lower risk for developing type 2 diabetes among Japanese subjects: Mikkabi cohort study. Bmj Open Diab Res. CA 3, e000147. 10.1136/bmjdrc-2015-000147 PMC4679813 26688736

[B35] Szeto H. H. Liu S. Soong Y. Wu D. Darrah S. F. Cheng F. Y. (2011). Mitochondria-targeted peptide accelerates ATP recovery and reduces ischemic kidney injury. J. Am. Soc. Nephrol. 22, 1041–1052. 10.1681/ASN.2010080808 21546574 PMC3103724

[B36] Teixeira R. B. Pfeiffer M. Zhang P. Shafique E. Rayta B. Karbasiafshar C. (2023). Reduction in mitochondrial ROS improves oxidative phosphorylation and provides resilience to coronary endothelium in non-reperfused myocardial infarction. Basic Res. Cardiol. 118, 3. 10.1007/s00395-022-00976-x 36639609 PMC9839395

[B37] Terao J. (2023). Revisiting carotenoids as dietary antioxidants for human health and disease prevention. Food Funct. 14, 7799–7824. 10.1039/d3fo02330c 37593767

[B38] Wang Y. Yang F. Liu T. Zhao C. Gu F. Du H. (2024). Carotenoid fates in plant foods: chemical changes from farm to table and nutrition. Crit. Rev. Food Sci. 64, 1237–1255. 10.1080/10408398.2022.2115002 36052655

[B39] Wu J. Shang H. Zhang A. He Y. Tong Y. Huang Q. (2023a). Antioxidant nanozymes in kidney injury: mechanism and application. Nanoscale 15, 13148–13171. 10.1039/d3nr01954c 37547960

[B40] Wu Q. Guan Y. B. Zhang K. J. Li L. Zhou Y. (2023b). Tanshinone IIA mediates protection from diabetes kidney disease by inhibiting oxidative stress induced pyroptosis. J. Ethnopharmacol. 316, 116667. 10.1016/j.jep.2023.116667 37257702

[B41] Xiao L. Xu X. Zhang F. Wang M. Xu Y. Tang D. (2017). The mitochondria-targeted antioxidant MitoQ ameliorated tubular injury mediated by mitophagy in diabetic kidney disease via Nrf2/PINK1. Redox Biol. 11, 297–311. 10.1016/j.redox.2016.12.022 28033563 PMC5196243

[B42] Zhang Y. Mao H. Li Y. Xiong Y. Liu X. Wang L. (2023). β-Cryptoxanthin maintains mitochondrial function by promoting NRF2 nuclear translocation to inhibit oxidative stress-induced senescence in HK-2 cells. Int. J. Mol. Sci. 24, 3851. 10.3390/ijms24043851 36835262 PMC9963668

